# In-Vitro Transcription analysis of NS5A from HCV-3a circulating in Pakistani patients with chronic hepatitis C and their differential response to antiviral therapy

**DOI:** 10.12669/pjms.335.12973

**Published:** 2017

**Authors:** Shameem Bhatti, Sobia Manzoor, Fahed Parvaiz, Javed Ashraf, Farakh Javed

**Affiliations:** 1 Shameem Bhatti, Department of Healthcare Biotechnology, Atta-ur-Rahman School of Applied Bio-Sciences, National University of Sciences and Technology (NUST), Islamabad 44000, Pakistan; 2 Sobia Manzoor, PhD, Department of Healthcare Biotechnology, Atta-ur-Rahman School of Applied Bio-Sciences, National University of Sciences and Technology (NUST), Islamabad 44000, Pakistan; 3 Fahed Parvaiz, Department of Biosciences, COMSATS Institute of Information Technology, Park Road, Chak Shahzad, Islamabad 44000, Pakistan; 4 Javed Ashraf, University of Eastern Finland – Kuopio, Finland; 5 Farakh Javed, University of Haripur (UOH), Khyber Pakhtunkhwa 22620, Pakistan

**Keywords:** HCV genotype, NS5A, Pakistan, Viral Load, peg-IFN therapy

## Abstract

**Objective:**

Mutations in HCV nonstructural protein 5A (NS5A) play a vital role in virus resistance. The aim of this study was to develop a correlation between NS5A mutations (genotype 3a) and virological response towards interferon alpha (IFN-α) plus ribavirin therapy.

**Methods:**

In this study, which was conducted from 09-02-2013 to 25-11-2015 in the rural area of Province Sindh – Pakistan, total patients’ responses to peg-IFN therapy were investigated. Patients were given peg-IFN therapy for 24 to 48 weeks and categorized as sustained virologic responders (SVR) or non-responders (NR) to HCV infection. HCV NS5A region (2215-2335) of genotype 3a was identified in both responders and non-responders.

**Results:**

Twenty-four NR with 24 SVR isolates showed significant mutations within the nonstructural protein 5A region in HCV genotype 3a. The New Zealand (NZL1) (GenBank D17763) differences were observed by using gene. The ISDR mutations for nonstructural protein 5A in non-responders have been reported as a possible explanation of HCV interferon resistance.

**Conclusion:**

Based on these results, it is suggested that decreased SVR is caused by the increased mutations in nonstructural protein 5A sequences. When the sequence outside the Protein kinases R binding domain (PKRBD) (2281–2335) was examined, significant differentiations were observed among the SVR and NR classes at few amino acid strains.

## INTRODUCTION

Hepatitis C Virus (HCV) is a positive single-stranded enveloped RNA virus with a genome size of approximately 9.6 kb.^1^ It is a hepatotropic virus that targets hepatocytes and is known to be the main causative agent of chronic hepatitis worldwide.^2^ Interferon (IFN) therapy for HCV chronic hepatitis is highly variable efficacy. The majority of patients do not obtain Sustained Virologic Response.^3^,^4^ Previous studies indicated that IFN mediated response is correlated to several host factors such as age, ethnicity, transmission of blood and blood borne products, duration of infection, iron deposition and liver fibrosis.^5^ Some other studies suggest that IFN mediated response is related to HCV factors such as genotype^6^,^7^ viral load^8^ and quasispecies.^9^ An HCV nonstructural protein 5A quasispecies pattern has been extensively associated with interferon resistance.^10^

As in previous studies from different countries, the use of sequence analysis of HCV nonstructural protein 5A coding region has shown specific domains within NSA region that differentiate in order to relate with the result of IFN therapy.^11^

Similarly, various reports have noted viral resistance in chronic HCV patients in different regions of Pakistan. Nevertheless, the increasing frequency of this epidemic disease accompanied by a decreased response to pegylated interferon therapy in rural areas, especially in the interior Sindh of Pakistan, is a great mystery. This study aimed to analyze the effect of the HCV nonstructural protein 5A sequence variation in peg-IFN combination therapy among responders and non-responders.

## METHODS

This study was conducted from 09-02-2013 to 25-11-2015 in the rural area of Province Sindh - Pakistan. The blood samples of HCV infected patients were sent to the diagnostic laboratory of Gambat Institute of Medical Sciences (GIMS), Gambat City, District Khairpur Mirs for liver function test (LFT), CBC and qualitative PCR for confirmation of HCV. HCV genotype and viral titer in blood were determined at the Viral Hepatitis Laboratory, Atta-Ur-Rahman School of Applied Biosciences (ASAB), National University of Sciences and Technology (NUST), Islamabad. Amplification of HCV NS5A and its sequences were performed at the Division of Infectious Diseases, Burnett-Womack Clinical Sciences Building, Chapel Hill, North Carolina, America. It included 212 patients infected with HCV, ranging in age from 20 to 80 years. Of 212 patients, 12 discontinue the therapy and among 200 patients 140 patients were diagnosed with HCV genotype 3a. The study was reviewed and approved by the ethical committee of Gambat Institute of Medical Sciences, Pakistan in association with ASAB, Department of National University of Sciences and Technology, Islamabad, Pakistan for blood sampling. Compulsory permissions were taken from all patients before proceeding to collect their blood samples.

Patients received recombinant Peg-IFN-Alpha at 180mcg/mL one time weekly with or without ribavirin doses for 6 to 12 months. Patients were tested for HCV RNA together with ALT and CBC levels during and after the course of therapy. In this study, patients were assessed for side effects of therapy and effectiveness of therapy, and they were evaluated with ALT and serum HCV RNA levels after 12, 24, and 48 weeks. Peg-IFN and ribavirin doses varied according to the weight, platelets, white blood cells counts, and hemoglobin level of each patient. The viral genotype was confirmed with genotypic specific primers according to the manufacturer’s protocol in the Ohno et al., with some modification.

Viral RNA was extracted from 140 µL of the each serum sample by using QIAamp Viral RNA extraction kit (Qiagen, USA) according to the procedure given in the kit protocol.

Viral load quantifications were done on Rotor-GeneTM 3000 (Corbett Research, Australia) real-time PCR system using aj Roboscreen AnylyticaGena (Gmb Germany) quantification modules. Primers and reaction conditions were first optimized for then on structural protein 5A viral gene on nested PCR the Bio-Rad C1000 thermal cycler (California, USA).

After amplification of nonstructural protein 5A by nested PCR, 20 μL each of the PCR residue then analyzed on 2% Agarose Gel was purified from Agarose Gel by a Gel Extraction Kit (Qiagen, USA) by strictly following the manufacturer’s protocol. The final evasion was made in 30 μl of Buffer EB (Qiagen, USA). Finally, purified DNA was analyzed on Thermo Scientific Nano Drop™ 2000c (IL, USA) and the purified amplified product was used for nonstructural protein 5A sequencing. Forward and reverse strands sequences were performed on an automated sequencer Applied Biosystems 310 DNA Sequencer (CA, USA) following the manufacturer’s protocol.

Sequence and Analysis - Nonstructural protein 5A sequences from patients infected with HCV were submitted to the National Center for Biotechnology Information. Amino acid sequences of the nonstructural protein 5A viral protein were aligned using Clustal W software.^12^ The nonstructural protein 5A 2215-2280 amino acid sequences were aligned with reference sequences such as NZl1 strain (D17763).

The data were analyzed using SPSS version 17. Assessment among responders and non-responder, Fisher’s exact test was practiced and P ≤ 0. 05 were measured as significant. The t-test was done to measure different HCV parameters.

## RESULTS

One hundred forty patients of HCV genotype 3a were analyzed and grouped into three different categories depending upon patient’s response towards standard HCV therapy. To plan for the dosage and the duration of therapy and to estimate the likelihood of a response, all patients infected with HCV underwent HCV genotyping prior to therapy. Therapy was pegylated interferon alfa 2a given subcutaneously for 24 or 48 weeks. 120 patients were given ribavirin and pegylated interferon. Twenty patients were given only pegylated Interferon therapy. Patients were categorized into one of three groups: therapy- naïve patients (before treatment), patients with sustained virologic response, and non- responders.

The results indicated that 109 patients (78%) were SVR and 31 (22%) were NR out of 140 chronically infected HCV patients of genotype 3a. SVR was defined as undetected HCV after 24 weeks of therapy.

Among patients, 66 (84%) males achieved sustained virologic response and 12 (16%) non-sustained virologic response while females achieved 43 (69%) sustained virologic response and 19 (31%) non-sustained virologic response. The mean age of the non-SVR was 46 years (31–65 years), which was higher than the sustained virologic response group’s mean age of 35 years (18–50).

Among 50 patients 02 patients did not show any mutation in NS5A. Sequences were obtained and searched for homology using Basic Local Alignment Search Tool (BLAST) in the nucleotide repository of National Center for Biotechnology Information (NCBI) database. To develop a consensus nucleotide sequence, each of the nonstructural protein 5A genes was aligned with the reference sequence using a clustal W2 sequencer viewer.

NS5A specific domain encoding ISDR and PKRBD (a 579bp fragment) was ranged to examine the dissimilarity in both groups of SVR and non-SVR mutations. These ranges were more classified into three residues; PKRBD, ISDR, and outside PKRBD. Amino acid ranges of the PRKBD were sequenced with the already available ranges of genotype 3a.

**Fig.1:**
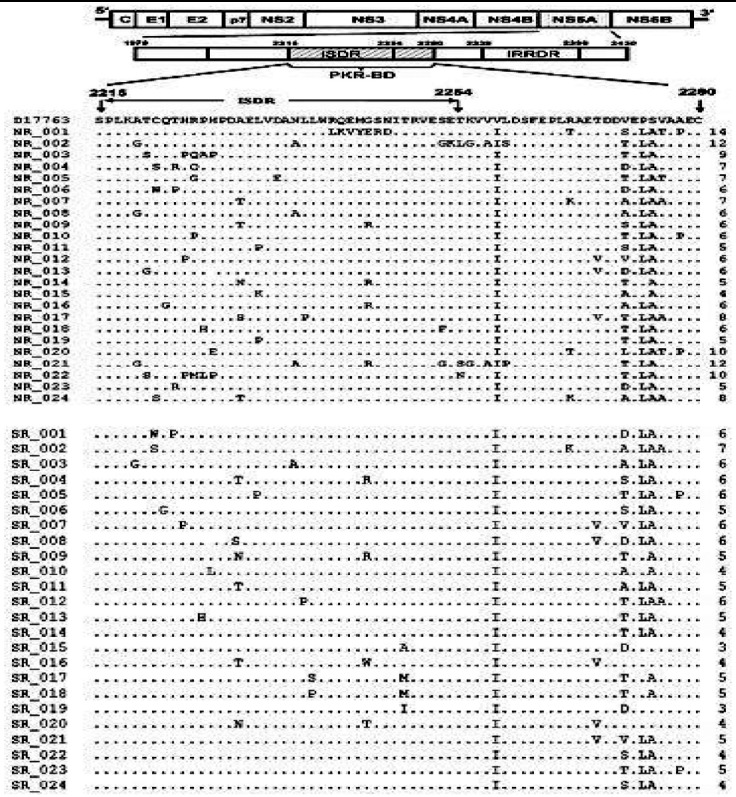
NS5A ISDR-PKRBD of HCV 3a mutation. Amino acid alliance were done compared with published sequences NZL1 (D17763).

From NS5A 2215-2280 sequences, mutations in both regions found from 00–07 in SVR (average = 04) 00–14 (average = 06) in non-SVR. Thus, mutations varied among non-responder and responders. These results indicated a clear dissimilarity in mutations among SVR and non-SVR in ISDR and PKRBD (Fig.1).

**Fig.2:**
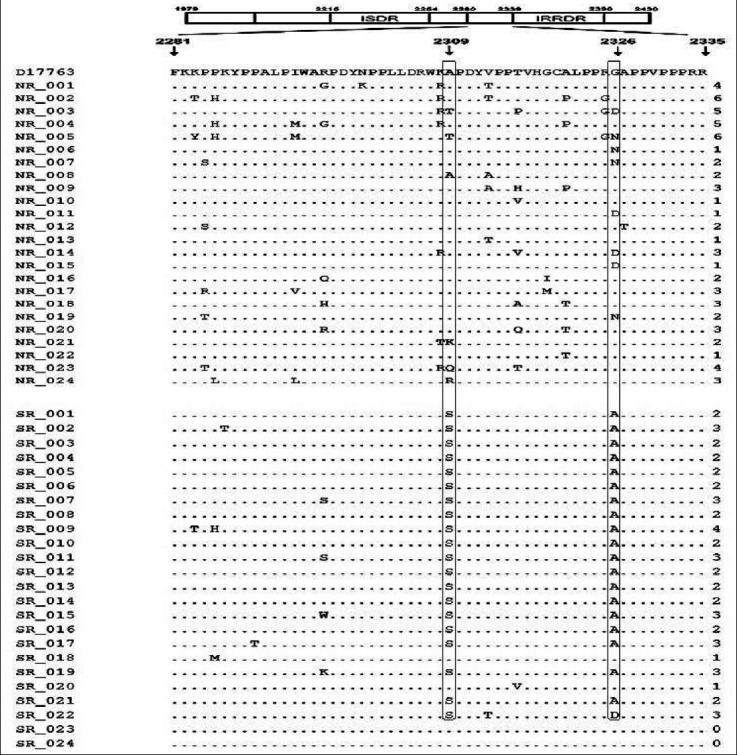
Association of NS5A sequence outer side the PKR binding site. Differences was found between groups (SVR and Non-SVR) at few amino acid position, 2309 aa Ala to serine and 2326 aa Gly to Ala. By using Student’s t-test, statistically significant difference was found (p ≤ 0.05).

Similarly, the range homology outer surface PKRBD9 (2281-2335) domain was analyzed between SVR and NR groups. It was observed that alanine (Ala) is replaced by serine (S) residue significantly at position 2309 as compared to the reference strain (Fig.2).

**Fig.3:**
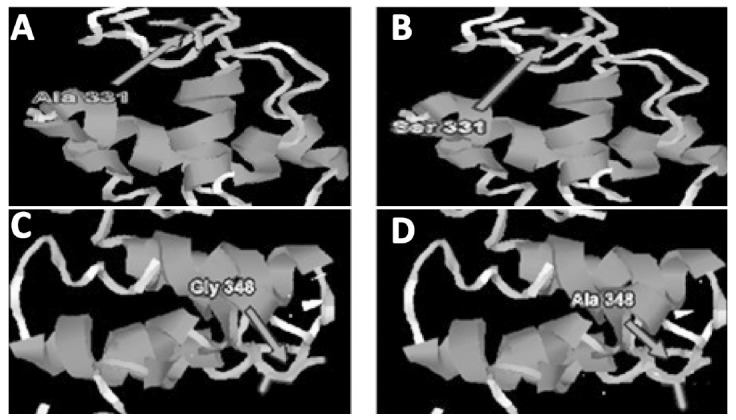
Mutations within PKRBD of NS5A protein were studied. Two main mutations was found at position 2309 (Ala to Ser) and 2326 (Gly to Ala). (a) Native NS5A structure with Ala at position 2309 of protein. (b) Ala is non-polar and hydrophobic than the Serine that is polar one. The primary modification at position 2309 (Ala to Ser) alters a OH group within the side chain of the mutant amino acid. The native residue (Ala) is non-polar and more hydrophobic than the mutant, which is a polar one. (c) Native NS5A structure with Gly situated at 2326 protein. (d) Mutation at location 2326 (Gly to Ala). The native Glycine is smaller and bendable than the Alanine. It is possible that Glycine at their position to change structure easily or to make facilitate protein movement.

While above mentioned mutation was analyzed among both groups (SVR and non-SVR), a clear dissimilarly in mutation among both groups was noticed. As well as, there is a replacement of glycine (Gly) with asparagine (Asn) and alanine (Ala) at position 2326. These mutations were higher in one group (SVR) highlighting few links in the direction of removal of virus after treatment with pegylated interferon therapy (Fig.3).

Mutations identified in NS5A sequences in ISDR with the percentage of SVR (24%) and non-SVR (76%). There was a clear dissimilarity in mutations among both groups (SVR and Non-SVR) including ISDR and PKBD (*P=<0.5). Mutations identified into NS5a PKRBD the percentage of SVR (69%) and NR (33%).

## DISCUSSION

HCV nonstructural protein 5A plays a critical role in HCV replication and particle assembly.^13^ Previous studies reveal that change of a single amino acid can intensely boost the efficiency by 70 to 500 times for colony formation.^8^,^14^ However, reports of several studies suggest that changes in amino acid orders nonstructural protein 5A of the hepatitis C virus are related to the viral load, genotypes, and outcome of IFN therapy.^15^,^16^

Contrary to an Indonesian study^17^, our results are showing high serum viremia with a significant number of mutations. The 579 bp fragment of the HCV NS5A region covers the interferon sensitivity determining region and the PKRBD. The PKRBD of NS5A is a 63 amino acid sequence in which the interferon sensitivity determining region is composed of the first 40 amino acids. This domain has been reported to be involved in the interaction with protein kinase R, which inhibits dimerization of protein kinase R and stops its antiviral activity, whereas the interferon sensitivity determining region has been associated with resistance to IFN combination therapy.^8^

This study reveals that increased mutations in HCV nonstructural protein 5A interferon sensitivity determining region domains lead to blockage of anti-viral pathways and prevent hepatocytes from undergoing apoptosis, which is in accordance with the previous study.^18^

Previously, very few studies on HCV genotype 3a patients in Pakistan showed any association of the NS5A-ISDR mutation in Responders as well as Non-Responders. These studies were conducted only in urban areas. Therefore, the results showed significant association of mutation because of differential mutation may be due to differences in geographic regions, ethnicity, or races. Therefore, further studies need to be performed in rural areas to elaborate on HCV therapy resistance to this epidemic disease. Various other factors such as race, ethnicity, and age have been related to IFN response in patients infected with HCV.^19^

## CONCLUSION

These results suggest that patients with lower viremia titers, slightly increased ALT levels and a higher platelet ratio before the start of therapy have a significantly enhanced response rate compared to those patients with higher viremia titers and a high rate of mutations in NS5A-ISDR in HCV. Furthermore, when a range outer side of PKRBD (2281-2335) was analyzed major dissimilarities were found among two groups (SVR and Non-SVR) at in the region of amino acid i.e. presence of Ala & Ser (outside PKRBD domain of NS5A) showed significant association with the clearance of hepatitis C virus.
